# Regulation of Collagen I and Collagen III in Tissue Injury and Regeneration

**DOI:** 10.26502/fccm.92920302

**Published:** 2023-01-20

**Authors:** Drishtant Singh, Vikrant Rai, Devendra K Agrawal

**Affiliations:** 1 Department of Translational Research, College of Osteopathic Medicine of the Pacific Western University of Health Sciences, Pomona, California 91766 USA

**Keywords:** Collagen I, Collagen III, Col I/Col III ratio, ECM Remodeling, Tissue Injury And Healing

## Abstract

The structure of connective tissues including cartilage, tendons, and ligaments as well as many organs, like the skin, heart, liver, kidney, lungs, blood vessels, and bones, depend on collagen. The bulk of the network of structural proteins that make up the extracellular matrix of the heart is composed of collagen type I and type III, which provide structural support for the muscle cells and are crucial for cardiac function. The prognosis and progression of a disease or diseased state may be significantly impacted by the upregulation or downregulation of the collagen types, particularly Col I and Col III. For example, increasing Col I protein levels may impose increasing myocardial stiffness, impairing the diastolic and systolic function of the myocardium. Collagen I is a stiff fibrillar protein that gives tensile strength, whereas Col III produces an elastic network that stores kinetic energy as an elastic rebound. These two collagen proteins have distinct physical properties in nature. Therefore, the control of Col I and Col III as well as the potential relevance of the Col I/Col III ratio in many biological processes serve as the foundation for this comprehensive review article.

## 1. Introduction

**Figure 1:**
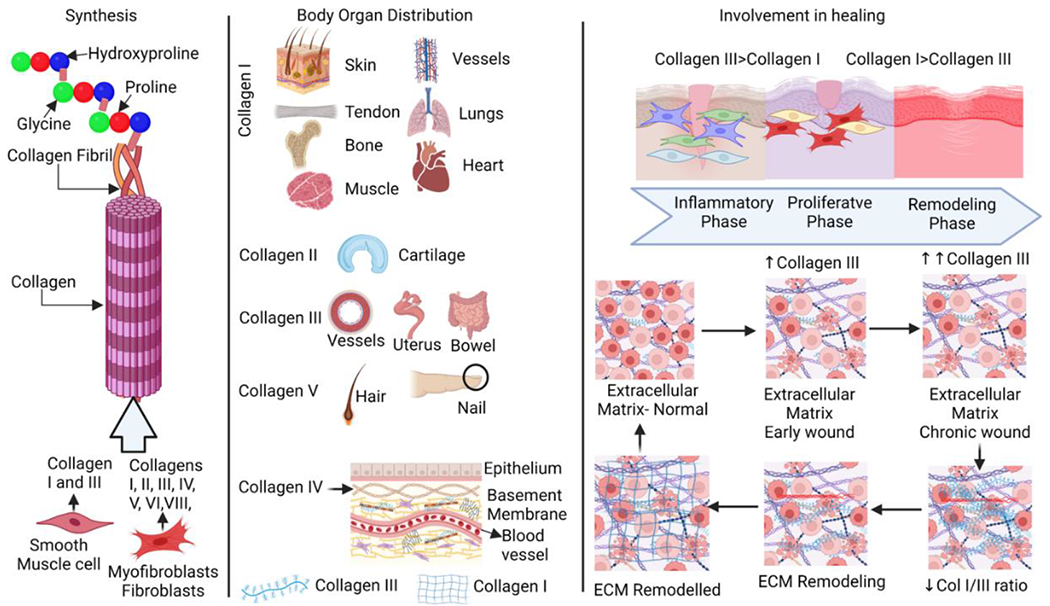
Collagen synthesis, distribution, and ECM remodeling. Collagen is synthesized by crosslinking of amino acids hydroxyproline, proline, and glycine, Crosslinking of these amino acids results in the formation of collagen fibrils and finally collagens. Collagens are differentially expressed in various body parts and pertain stability, elasticity, and strength along with the involvement in various physiological processes. Changes in collagen distribution during wound healing and ECM remodeling during injury where the ratio of collagen I and collagen III is altered with a higher amount of collagen III during healing while increased collagen I in a healed wound.

Collagen is an essential component of the structure of connective tissues such as cartilage, tendons, and ligaments as well as several organs including skin, heart, liver, kidney, lungs, blood vessels and bones [1]. Collagen belongs to a class of fibrous protein family that is a part of the extracellular matrix (ECM) [2]. It consists of three alpha chains that coil around one another to form the collagen fibers. The integral structure of the collagen and its type are determined by variations in amino acid sequence of the chain [3]. Collagen may be classified as interstitial collagen, basement membrane collagen, and peripheral collagen depending on where it is found in the body. There are now more than 30 different kinds of collagen known and documented. Three left hand spirals (proline II), intertwined and joined to one another to create a long and robust right hand spiral structure, make up the normal collagen molecule, commonly known as the triple helix [4]. In extensible connective tissues including the skin, lungs, and vascular system, collagen type I (Col I) is usually seen in association with type III collagen (Col III). The main types of collagens that are present in ECM are collagen type I and III, however collagen types IV, V, VI, and VIII are also present in ECM [5,6] (Figure 1). Collagen type I builds a scaffold with thick fibers that have a low turnover rate. The maturation of collagen type I, however, depends on collagen type III, which produces thin, less durable fibers with a high turnover [7]. Also, the collagen types I, II, and III are the most prevalent fibrillar collagens. Skin, tendons, vasculature, lungs, heart, and other organs all contain collagen type I [8–11], which also makes up most of the organic material in the calcified tissue of bones and teeth [12] (Figure 1). However, reticular fibers are made of collagen type III which are often seen along with collagen type I.

Collagen type I and collagen type III, which serve as structural support for the muscle cells and play a significant role in cardiac function, make up most of the network of structural proteins in the ECM of the myocardium [13]. The protein levels of collagen in the myocardium have been found to be changed in dilated cardiomyopathy (DCM), which is largely defined by an accumulation of Col I [14]. Due to their various mechanical qualities, the ratio of collagen types in the heart is important [15]. Previous study used electron microscopy and immunohistochemical techniques to show increased quantity of collagen fibers in end-stage DCM [16]. Another biochemical investigation revealed a rise in the absolute levels of both Col I and Col III [17]. However, several studies showed contrasting results where a significant increase primarily in thin collagen fibers and a reduction in thick collagen fibers were observed in the heart. In a recent report, patients who had myocardial infarction and had coronary artery bypass graft had considerably lower levels of collagen type III in the aortic wall samples than those who had stable angina [18]. Also, collagen type III is more vulnerable to changes in the local vascular wall leading to the development of unstable atherosclerotic plaque because it is thin, less stable, and more likely to experience inflammatory responses [19–22]. The increase in collagen gene expression is regulated by the promotors of Col I and Col III genes containing regulatory elements related to transforming growth factor beta (TGF-β) [23]. Since the Col I promotor also contains several binding sites for specificity protein 1 (SP-1) that are not found in the Col III promotor, the differential gene expression levels of Col I and Col III might rely on their respective promotors [24]. The difference in the levels of Col I and Col III may depend not only on the differential gene expression but also on differential degradation of both proteins. Two different matrix metalloproteinases (MMPs) are described as being active in degrading collagens. MMP-1 is synthesized by fibroblasts and shows equal affinity for Col I and Col III degradation. MMP-8, however, which is synthesized by neutrophils, has a higher affinity for Col III. Therefore, either differential activities or differential expression levels of MMP may also contribute to changes in myocardial Col I and Col III content, in addition to the changes in gene expression [25, 26]. Nonetheless, Col I and Col III are essential components of the myocardium, maintaining its structural and functional integrity. The upregulation or downregulation of the collagen types, specifically Col I and Col III, might have a major impact on the prognosis and development of a disease or diseased state such as increasing Col I protein levels might impose increasing myocardial stiffness, compromising diastolic and systolic function of the heart [12]. The physical characteristics of both the collagen proteins are different in nature where Col I represent a stiff fibrillar protein that provides tensile strength, in contrast to Col III which forms an elastic network storing kinetic energy as an elastic rebound. Therefore, this critical review addresses the regulation of Col I and Col III and the potential significance of Col I/Col III ratio in biological activities.

## 2. Collagen Metabolism

The collagen network is a metabolically active structure with a collagen turnover, which most likely occurs between 80 and 120 days, is determined by the equilibrium between collagen production and breakdown [27]. The changes in the collagen number depends upon fibroblasts, particularly the fibroblasts that have differentiated into myofibroblasts, the phenotype contributing to collagen turnover [28]. These cells react to mechanical strain, autocrine and paracrine substances produced locally (such as TGF-β and growth factors like angiotensin II), and hormones (such as aldosterone) received from the circulation. The activity of fibroblasts and myofibroblasts is also influenced by a variety of proinflammatory cytokines which are released by monocytes and macrophages [29]. The ability of these cells to produce and secrete fibrillar collagen precursors, specifically the two more prevalent subtypes of procollagen found in the heart, (Col I and III), as well as enzymes that convert procollagen precursors into mature collagen capable of forming fibrils and fibers (such as procollagen proteinases and lysyl oxidase) is dependent upon the changes in their rates of proliferation and migration and modifications in response to all the aforementioned factors [30,31]. Numerous clinical investigations of candidate biomarkers of collagen metabolism fall into two categories: (i) biomarkers related to the synthesis of collagen molecules that form new collagen fibers, and (ii) biomarkers related to the degradation of collagen molecules that integrate the old fibers [32].

### 2.1 Collagen Biosynthesis

Cells such as fibroblasts (resident and myeloid cell transformed fibroblasts) are the primary source of freshly generated collagen in the healing wound [33]. The biosynthetic activities of fibril-forming collagens are the most widely investigated of all collagens, necessitating the temporal and spatial coordination of several biochemical processes. In the endoplasmic reticulum, the nascent collagen molecule is converted into pro-collagen by removing the signal peptide from its N-terminus after transcription [34]. The creation of the triple-helical structure typical of collagens is caused by the hydroxylation and glycosylation of amino acid residues [35]. The pro-collagen triple-helical structure is stabilized in the Golgi apparatus for further processing and maturation and formed into secretory vesicles that are extruded into the extracellular space where the pro-collagen is enzymatically converted into tropocollagen [36]. Covalent cross-linking is used to assemble the final collagen fibril. This cross-linking mechanism is responsible for the mechanical characteristics (elasticity and reversible deformation) of fibrillar collagens [37]. Among these crosslinks are the cystine-cystine disulfide bonds, transglutaminase cross-links, cross links related to advanced glycation end (AGE) products and cross-links created through the lysyl oxidase pathway which are reducible and mature [38, 39]. The degradation of cross-linking varies depending on the kind of collagen and the tissue environment, resulting in a multi-layered hierarchical structure [40]. Mature cross-links increase shear stress resistance. AGE-specific cross-links lead to increased collagen stiffness in aged tissues [41]. Fibroblasts and myofibroblasts produce procollagen types I and III in the form of a triple-helix procollagen precursor with terminal propeptides (Figure 1). These propeptides are cleaved by specific procollagen proteinases, allowing the resulting collagen molecule to be integrated into the expanding fibril. The propeptides are released into the bloodstream and are detectable in the blood [42, 43]. Collagen propeptides may serve as indices of collagen synthesis if they are cleaved in every molecule of collagen and if the number of propeptides measured in the circulation is proportionate to the amount of collagen generated [44, 45]. This is true for the procollagen type I carboxy-terminal propeptide (PICP) and, most likely, the procollagen type I amino-terminal propeptide (PINP) [46, 47]. There is a one-to-one stoichiometric relationship between collagen type I synthesis and PICP secretion. However, during the conversion of procollagen type III into collagen type III, the carboxy-terminal and amino-terminal propeptides of collagen type III (PIIICP and PIIINP, respectively) are not fully cleaved, remaining to some extent in the final fiber and thus also being released during fiber degradation [48, 49]. As a result, there is some flexibility in the stoichiometric ratio between the amount of collagen type III made and the amount of PIIICP and PIIINP released [43].

### 2.2 Collagen Degradation

**Figure 2:**
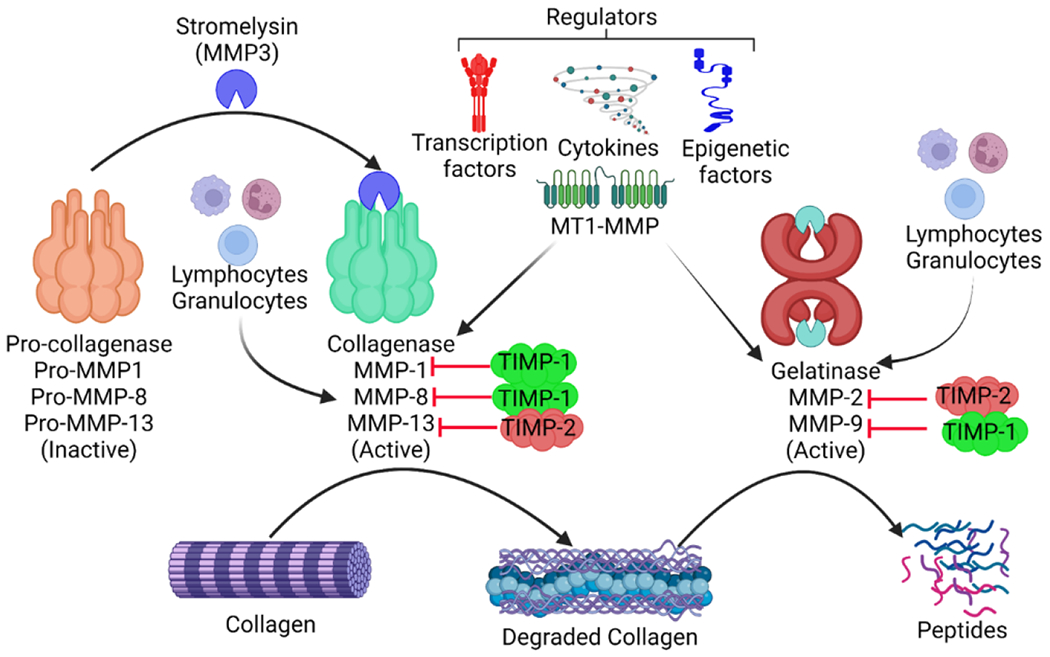
Regulation of collagen degradation: Collagen degradation is regulated by matrix metalloproteinases (MMPs) which in turn are regulated by tissue inhibitors of matrix metalloproteinases (TIMPs) and membrane type 1 matrix metalloproteinase (MT1-MMP). MMPs are secreted by lymphocytes and granulocytes and their secretion is regulated by cytokines, transcription factors, and epigenetic factors. Collagenases are secreted in an inactive form and get activated via stromelysin. This suggests that the activation and secretion of MMPs are tightly regulated processes.

The degradation of collagen is implicated in inflammation, angiogenesis, and re-epithelialization, which are all controlled by complicated molecular processes [50]. During the inflammatory phase, soluble collagen fragments attract immune cells such as macrophages, which patrol the wound for the elimination of microorganisms and devitalized tissue [51]. This facilitates the shift to the proliferative stage. During this stage, collagen fragments act as powerful angiogenic signals, promoting the formation of new blood vessels. Collagen also promotes keratinocyte migration, which aids in wound re-epithelialization [52, 53]. Extracellular and intracellular mechanisms control degradation. Membrane-bound and secreted proteolytic enzymes are involved in the extracellular mechanism [54]. Internalization of intact collagen fibrils and fragmented collagen (through phagocytosis, macropinocytosis, or endocytosis), followed by enzymatic breakdown, is involved in the intracellular mechanism. Pathological disorders such as fibrosis are caused by defects in the controlled turnover of collagens [55]. The activity of proteolytic enzymes at various phases of healing of a tissue guide the remodeling of healed tissue [55]. The MMPs and serine proteases are two major enzyme families to degrade proteins. The synthesis and secretion of these enzymes are carefully controlled and connected with certain cellular subtypes [1, 56]. Collagenases and gelatinases, which destroy intact and damaged fibrillar collagen, respectively, are MMPs that are important for collagen turnover during the healing of various tissues. MMP-1 (also known as collagenase-1) and MMP-8 (collagenase-2) preferentially cleave collagens I and III, whereas MMP-9 (gelatinase) degrades collagen IV [57] (Figure 2). Collagenolytic enzymes can detect, bind, unwind, and break the constituent strands of the triple helix, according to extensive study. This great specificity is thought to be generated by the main and super-secondary structures of collagen. MMPs are responsible for both physiological (development and tissue repair) and pathological (tumorigenesis and metastasis) activities. They also aid in the release of bioactive fragments (also known as matricryptins) from full-length collagens, such as endostatin and tumstatin [58]. These pieces precisely direct blood channel pruning, allowing for the restoration of tissue architecture during healing [59, 60]. Neutrophil elastase, a serine protease, contributes to the same mechanism. Therefore, the injury and healing of a tissue requires a tightly regulated balance of enzyme activity and inhibition. Imbalances in these enzyme levels can lead to severe conditions of a diseased state.

Chronic wounds are exacerbated by wounds infected with bacteria that generate collagen-degrading enzymes. The MMP family of enzymes plays a central role in collagen fiber degradation and can be stopped in their tracks by interacting with tissue inhibitors of metalloproteinases (TIMPs) (TIMP-1 to TIMP-4) [61, 62] (Figure 2). Collagen digestion begins when the peptide link after a glycine residue about 3/4 of the way from the amino-terminal end of the collagen molecule is hydrolyzed by interstitial collagenase (MMP-1), neutrophil collagenase (MMP-8) and collagenase-3 (MMP-13) [63, 64]. MMP-1 digests collagen type I, releasing a one-quarter carboxy-terminal telopeptide (CITP) that is present in the blood unprocessed by the immune system [65]. The amount of fibrillar collagen destroyed is directly linked to the amount of CITP released into the circulation, and the two processes have a stoichiometric ratio of 1:1. Thus, CITP may be used as a measure of collagen type I degradation that is MMP-1 dependent [66, 67]. MMP-2 and MMP-9, or gelatinases, further breakdown the amino-terminal telopeptide fragment released by MMP-1 from the collagen molecule [68, 69]. Matrikines, the fragmented matrix peptides generated by these enzymes, have biological actions in the control of collagen metabolism and angiogenesis. Collagen type I tripeptide glycyl-histidyl-lysine (GHL) is one such example that promotes collagen synthesis in fibroblasts [70]. There may be a redundant and cooperative role among some MMPs and matrikines, as it has been demonstrated that GHL increases MMP-2 production and secretion by fibroblasts in culture [71].

## 3. Significance of Col I and Col III Ratio and its Metabolic Regulation

**Figure 3:**
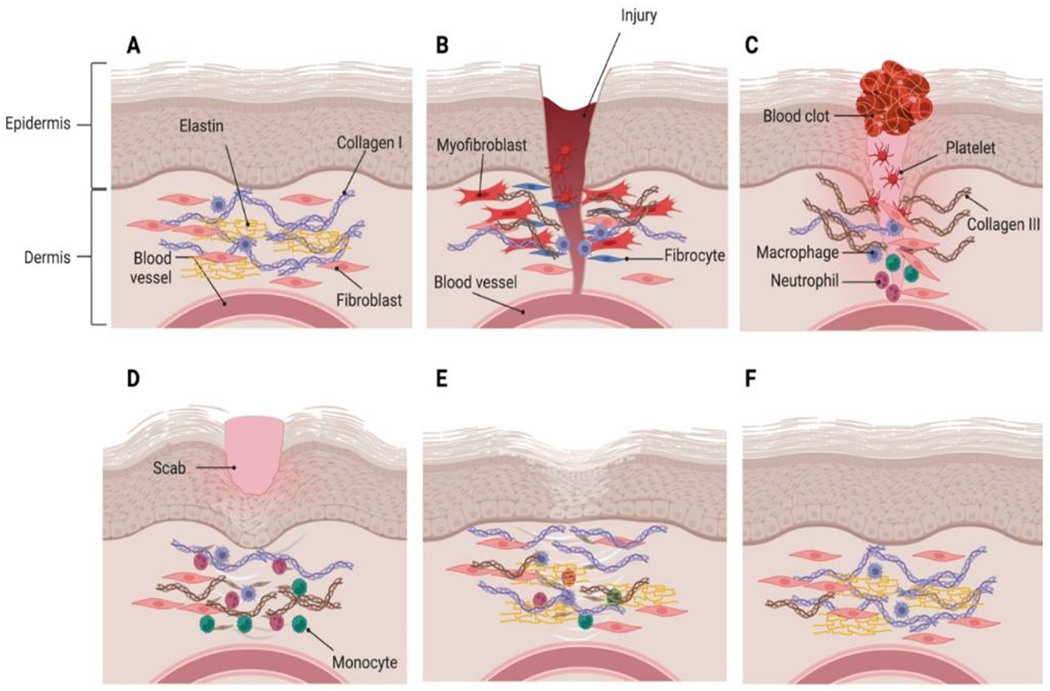
Different phases of an injury and wound healing process. A. Normal skin having epi- dermis and dermis. B. Injury and blood flow to the site of injury. C. After injury, a clot is formed to prevent blood loss. The macrophages promote tissue remodeling and col III level starts increasing at this stage. D. Contraction of wound and scab formation. E. The maturation of tissue results in formation of scar tissue because of remodeling of the ECM where col III is replaced by col I. F. Restoration of normal skin after wound healing.

The main regulators of collagen levels in tissue and cells (such as fibroblasts) include MMPs and tissue inhibitors of MMPs (TIMPs) which are also required for ECM homeostasis [72]. MMPs are proteolytic enzymes that breakdown ECM proteins, whereas TIMPs are MMP inhibitors that keep the production and degradation processes in check [72]. The alteration of regulated synthesis and degradation of collagen has been potentially linked to many diseases including various cardiovascular diseases. ECM is composed of a fibrillar network, a basement membrane, proteoglycans, and fibrous proteins such as fibronectins, collagens, elastins, fibrillins, and laminins [73]. They work together to keep border cells structurally coherent and stable. The ECM has also been linked to the transmission of critical biochemical signals required for appropriate tissue growth. ECM remodeling is described as a set of molecular, cellular, and interstitial changes that appear clinically as modifications in the size, mass, shape, and function of the heart after a stressful stimulus [74]. This process may be induced by inflammation, ischemia, cell migration, and other cellular processes [75]. Alterations in the morphology and function of heart ventricles can occur from disruptions in collagen metabolism, which in turn cause anomalies in the remodeling of the collagen network [76]. Collagen fiber buildup can develop when collagen production outpaces breakdown [77]. Different types of myocardial fibrosis, such as those that occur during repair and those that occur in response to damage, each contribute to ventricular hypertrophy and diastolic dysfunction [78, 79]. In contrast, ventricular dilatation and systolic dysfunction may result from the loss of the collagen scaffold and/or a weakening of the matrix due to a degradation-dominated cellular environment [80]. These two patterns may coexist to varying degrees within the same myocardium depending on the time course of the disease process and the localization, either diffuse or focal, of the injury [75, 81]. Ischemic heart disease, due to pressure overload, volume overload, and intrinsic myocardial disease or cardiomyopathy have all been hypothesized to involve the changes of the collagen network [80, 82]. Incorporating keratinocytes into a collagen matrix has also been reported to reduce inflammation and promote healthy epidermal growth. This suggests that collagen promotes cell mobility through the matrix, which in turn promotes a rapid wound-healing process. Figure 3 represents different phases of an injury and subsequent wound healing process involving an increase in the ratio of Col III:Col I.

### 3.1 Regulation of Col I/Col III in Cardiovascular Disorders

Myocytes and fibroblasts are supported by the comprising macromolecular network of fibers. The synthesis and degradation of collagen, a principal structural protein results from a balance of biochemical mediators, ischemia, stretch and inflammation [83]. Collagen type I and III are abundantly present in the myocardium. Collagen type I represent 85% of the cardiac collagen, has poor specificity, but confers tensile strength and resistance to stretch and deformation. On the other hand, type III is less abundant but more specific to the heart and confers resilience [84]. The fibrillar collagen in the myocardium serves as substrate for MMPs of which, MMP-1 has the highest affinity for fibrillar collagen and specifically degrades collagen I and III [85]. Interestingly, MMP-1 degrades ~40% of the newly synthesized collagen in various tissues. The net level of MMP-1 activity is dependent on the relative concentrations of active enzyme and metalloproteinase tissue inhibitors viz. TIMPs. In cardiac fibroblasts, the co-expression of MMP-1 and TIMP-1 is significantly regulated for maintaining the ECM architecture [86]. ECM remodeling is vital in a cardiac remodeling, hypertensive cardiac hypertrophy, dilated cardiomyopathy, and post-infarction healing [87]. The production of CITP, a telopeptide of pyridinoline crosslinks and a marker of collagen type I degradation results from the hydrolysis of collagen type I fibrils by MMP-1 [88]. Changes in ECM matrix turnover can result in cardiac fibrosis, a major determinant of diastolic dysfunction and pumping capacity. It also serves as a structural substrate for arrhythmogenicity and potentially contributes to the progression of congestive heart failure and sudden death [89]. Fibroblast stimulation, proliferation, phenotypic transformation is related with variable expression in MMPs which has a significant role in ventricular remodeling and thus, pathophysiology of hypertension, myocardial infarction, and heart failure [30]. The significant association of fibrous tissue with myocardial dysfunction and failure necessitates usefulness of non-invasive assessment of fibrosis as a clinically useful tool, particularly with the potential for cardioprotective and cardioreparative pharmacological strategies. The evaluation of cardiac collagen turnover using biological markers is a useful tool for monitoring “at a distance” cardiac tissue repair and fibrosis [49]. The assessment of serum peptides arising from the metabolism of collagen type I and III can provide insights to the extent of myocardial fibrosis [45]. Procollagen type I C-terminal propeptide (PICP) and amino terminal propeptides of type-I procollagen (PINP), and N-terminal type III collagen peptide (PIIINP) released with collagen type I or III molecules in a stoichiometric manner during collagen biosynthesis are considered potential markers of the process [90]. However, these markers are not specific to the myocardium. Predominantly in hypertension and diabetes affecting various organs, especially vascular tissues, in which procollagen fragments could be released; Querejeta et al. showed a correlation between myocardial collagen content and serum concentration of PICP in hypertension [91]. Recently, they reported secretion of serum PICP by the heart through the coronary sinus in patients with hypertensive heart disease [44]. The PIP/CITP ratio, an index of coupling between synthesis and degradation of collagen type I, was observed to be elevated in hypertensive patients with increased collagen accumulation in myocardial tissue compared to those with normal accumulation of collagen [92]. Thus, serum levels of PICP/CITP ratio serves as a marker of myocardial collagen accumulation. In dilated cardiomyopathy patients, it was reported that both, collagen volume fraction and collagen types I and III mRNAs in the myocardium were higher in the patients with an increased PICP/CITP serum level ratio compared to those with lower PIP/CITP ratio [93]. The findings support links of serum ECM markers to the heart ECM content, thus providing rationale for their use as potentially useful biomarkers of ECM remodeling in cardiac disease [94]. Furthermore, the potential usefulness of the MMP-1 and TIMP-1 serum levels ratio as a marker of myocardial collagen degradation, myocardial expression of MMP-1 and its tissue inhibitor TIMP-1, and the quantity and distribution of fibrillar collagen deposits have been assessed concomitantly to measure serum MMP-1 and TIMP-1 in the same patients with hypertension [95]. In hypertensive patients, the values of MMP-1 and TIMP-1 in coronary sinus blood were comparatively higher than in peripheral vein blood, but not in normotensive subjects [96]. Moreover, significant associations of MMP-1 and TIMP-1 in coronary sinus blood and peripheral vein blood in all hypertensive patients were observed [97]. The authors did not report any association of myocardial expression of MMP-1 and TIMP-1 with serological markers or the amount and distribution of fibrillar collagen [98].

### 3.2 Col I/Col III Ratio in Inflammation

Hemostasis and inflammation are both part of the inflammatory phase of wound healing. Injury to collagen stimulates the clotting cascade, resulting in a fibrin clot that stops the initial bleeding. Collagen I and IV fragments may operate as inflammatory mediators by serving as neutrophil chemoattractants, increasing phagocytosis and immunological responses, and regulating gene expression [58]. Inflammation is a key phase in the normal healing process, driving the proliferation of fibroblasts that generate collagen and ECM [99]. In proper wound healing, the resolution of inflammation in a timely way is equally crucial. Inflammation resolution is a dynamic process fueled by a mix of pro and anti-inflammatory reactions. According to one research that used a stabilized collagen matrix, collagen mounts a powerful and acute inflammatory response that is brief and fades quickly to allow wound healing to progress [100]. Furthermore, collagen has been shown to play a key role in creating an anti-inflammatory, pro-angiogenic wound macrophage phenotype through microRNA signaling [101].

### 3.3 Col I/Col III ratio in ECM Remodeling

Collagens are structural components of the ECM that help to maintain skin elasticity while also stabilizing growth factors and regulating cell adhesion and communication between cells and the ECM [101]. The adult wound heals with the production of a ‘typical’ scar throughout the healing process, as the damaged tissue undergoes remodeling over time. Scar tissue regains 50-80% of the initial tensile strength of normal skin but may be functionally inadequate [103]. The density, fiber size, and orientation of the collagen fibrils seem to be the primary difference between scarred and unwounded skin [52]. Angiogenesis, a vital component of both physiological (development, wound healing) and pathological (cancer) processes, is carefully controlled by the action of stimulators and inhibitors in a balanced manner. Collagens play a significant role in ECM remodeling, which provides vital support for vascular formation [104, 105]. Depending on the kind of collagen, it may either stimulate or prevent angiogenesis. A live multiphoton microscopy examination of in vitro neovessel formation revealed a dynamic modulation of collagen I that demonstrated early-stage remodeling of collagen fibrils moving to collagen condensation in later stages of development [103]. Collagen I have been shown to induce angiogenesis in vitro and in vivo by binding to certain integrin receptors [106]. The C-propeptide component of collagen I recruit endothelial cells, possibly inducing angiogenesis in wound healing. Proteolytic collagen fragments of collagen IV and XVIII, on the other hand, have anti-angiogenic activities (e.g., endostatin, arresten, canstatin, tumstatin, tetrastatin) [59]. These fragments decrease endothelial cell proliferation and migration and induce endothelial cell death, and thus prevent angiogenesis in a variety of clinical situations [1, 8,107].

### 3.4 Col I/Col III Ratio in the Skin and Wound

Collagen improves tissue mechanical strength and flexibility while also serving as a natural substrate for cellular adhesion, proliferation, and differentiation. The biofilm-mediated upregulation of MMP-2 via microRNAs in the wound creates a collagenolytic environment, sharply decreasing the collagen I/collagen III ratio and compromising the biomechanical properties of the repaired skin, potentially making the repaired skin vulnerable to wound recurrence [108]. A recent collagen structure and function mapping research revealed that in normal, wounded tissue, the collagen fibril is in a closed conformation that, when exposed to blood after injury, reveals cell- and ligand-binding sites that may aid wound healing [109]. Several recent studies go into depth into the roles of collagen in the skin and wounds [110, 111]. Several investigations have demonstrated that changes in collagen protein composition are mainly characterized by a considerable shift in the type I to type III collagen ratio [112, 113]. The relative ratios of type I and type III collagen fibers play a significant role in the control of fibrillogenesis and the determination of final fibril diameter and bundle architecture [114, 115]. From a pathophysiologic standpoint, mature type I collagen is primarily responsible for mechanical stability, while type III collagen that produces thin strands is mostly considered juvenile collagen of the early wound healing phase [116].

## 4. Conclusion

The change in expression of collagen type I and III is very crucial in tissue injury and ECM remodeling. Collagens can directly change the wound microenvironment, act as a scaffold for cell attachment and function, and provide biologically active principles or antimicrobials to help wounds heal. The type I and type III collagen have different properties that control how they perform their functions during different stages of a biological process. The type I collagen has a higher number of nonpolar residues due to which it can rapidly assemble the monomers to the side of the fibril to make it bigger. During the initial stages of tissue remodeling or injury, the expression of collagen III increases as it is more hydrophilic than collagen III and its fibril formation is slow and random. The further reorganization of collagen occurs by covalent cross-linking of the fibrils that leads to maturation of the collagen into complex structures and ultimately restores the tensile strength of the fibrils which is a property of Collagen I. Type I and type III collagens are essential for wound healing and tissue regeneration, with an increase in type III collagen synthesis during the early phases and an increase in type I collagen synthesis during the late stages. Understanding the role of type I and III collagens involved in ECM remodeling in tissue injury and regeneration, is essential for developing new cardioprotective strategies.

## 5. Future Directions

The alteration in Col I and Col III levels is an important process in tissue remodeling and wound healing. Many cross-sectional studies measured Col I and Col III peptides at a specific time point but the concentration and ratio of Col I and Col III should be studied at different time points during various biological processes. Furthermore, the altered ratio of Col I/Col III may not be specific to tissue injury or ECM remodeling and changes in their levels may represent a wide range of biological processes and/or pathological conditions. Fibrosis affects various organs, and the altered expression of collagen type I and III may originate from different organs such as heart, liver, lungs, bone, skin, or kidney. Therefore, the studies should be aimed to define the correlation of the altered expression levels of col I and col III in various tissues with their blood concentration along with the histological analysis to determine the differences in leakage from tissues. This could possibly help in detecting tissue specific novel targets that might have roles in the regulation of col I and col III during various biological processes. Future research into collagen gene regulation, gene switching, and the control of collagen synthesis and degradation is needed to learn more about how the collagen network is linked to tissue injury and regeneration and to find ways to stop, slow down, or reverse the abnormal remodeling of the collagen matrix.

## Data Availability

Not applicable since the information is gathered from published articles.
